# Influence on Growth and Physiology of *Campylobacter jejuni* Under Simulated Microgravity

**DOI:** 10.3390/pathogens15101028

**Published:** 2026-09-30

**Authors:** Roberta Garces, Alexis Ho, Madhusudan Choudhary, Todd P. Primm, Sofiane Gana, Anand B. Karki

**Affiliations:** Department of Biological Sciences, Sam Houston State University, Huntsville, TX 77340, USAmxc017@shsu.edu (M.C.); tprimm@shsu.edu (T.P.P.); sxg195@shsu.edu (S.G.)

**Keywords:** *Campylobacter*, microgravity, virulence genes, cell length, stress

## Abstract

*Campylobacter jejuni* is a major cause of gastrointestinal infections worldwide and commonly transmitted through the ingestion of undercooked, contaminated meat. Despite the extensive knowledge of pathogenic bacterium’s behavior on the Earth, there is no study with *C. jejuni* under spaceflight conditions. This study examines the influence of simulated microgravity on the growth, cell length, and virulence gene expression of *C. jejuni*. Microbial cultures were maintained under simulated microgravity using a Rotary Cell Culture System and compared with cultures grown under normal gravity conditions. No significant differences in bacterial growth were observed under simulated microgravity compared with normal gravity after 48 and 72 h of incubation. Cell length was shorter in both simulated microgravity and normal gravity (Falcon tubes) cultures than in the normal gravity (HARV) control, although differences were not significant based on mean cell-length values from biological replicates using a paired *t*-test. In contrast, mixed-effects analysis of individual cell-length measurements revealed significant differences between simulated microgravity and the normal gravity HARV control and between the normal gravity (Falcon tubes) and normal gravity (HARV) control conditions. Gene expression analysis using quantitative PCR was performed on selected virulence genes (*cadF*, *ciaB*, *cdtA*, *luxS*, and *flaA*) associated with adhesion, invasion, toxin production, quorum sensing, metabolism, and motility. This study found no significant differences in gene expression under simulated microgravity compared to normal gravity for the selected genes. Results indicated the limited influence of microgravity on *Campylobacter* physiology, but further investigation utilizing whole-genome transcriptomic and proteomic analyses is warranted to gain a deeper understanding.

## 1. Introduction

*Campylobacter jejuni* is one of the top leading bacterial foodborne pathogens all around the world, causing diarrheal illness, and remains a major cause of immunological disorder Guillain–Barr syndrome [1,2,3,4,5]. *C. jejuni* is a Gram-negative, rod-shaped, microaerophilic bacterium that is commonly transmitted to human hosts through contaminated retail meat products like raw poultry, chicken, pork, and beef [6,7,8,9]. The high prevalence of *C. jejuni* in retail meat products and increasing antimicrobial resistance among these pathogens are concerning issues for public health and food safety [10,11,12,13,14,15,16,17]. According to the Centers for Disease Control and Prevention (CDC), approximately 1.5 million cases of *Campylobacter* infection occur in the United States each year [18]. After colonizing the human intestinal tract, *C. jejuni* breaches the intestinal epithelial layer, resulting in abdominal pain, fever, diarrhea, and malaise [6,8,19,20]. Optimum growth conditions for *Campylobacter* include a microaerobic environment (5% O_2_ and 10% CO_2_) and 37–42 °C temperature. Understanding the environmental conditions and stressors that influence the survival and molecular mechanisms of *C. jejuni* is essential for reducing campylobacteriosis and improving food safety [13,14,15,16].

As space exploration expands, it is increasingly important to understand how microorganisms, including pathogenic bacteria like *C. jejuni*, respond to environments with gravitational forces different from the earth [21,22,23,24]. Microorganisms have been shown to alter their behavior in ways that may cause health risks to astronauts during spaceflight [21,22,23,24]. Studies on the influence of variable gravitational forces are becoming increasingly relevant, as ambitious space exploration and the prospect of human habitation on the Moon and other celestial bodies appear increasingly feasible [25,26]. Simulated microgravity can be generated using a rotating vessel which maintains cells in continuous suspension [21,27]. In this study, a Rotary Cell Culture System (RCCS-4 H; Synthecon Corporation) equipped with High-Aspect-Ratio Vessels (HARVs) was used to examine *C. jejuni* under simulated microgravity conditions, similar to those used in our previous study investigating the influence of simulated microgravity on *Acidovorax* sp. 1,608,163 and multiple other studies [21,24,28,29,30].

Bacterial responses to microgravity vary widely among bacterial species [21,24,31]. Previous studies have reported that microbial growth, metabolism, chemotaxis, motility, virulence gene expression, and expression of other genomic determinants are affected by microgravity [21,22,23,24,31,32,33]. Although no universal response towards them has been identified yet, some microorganisms experience physiological stress in low-gravity environments [31]. Microgravity may cause alterations in primary metabolic pathways, including those involved in nucleotide, amino acid, and organic acid metabolism; energy production; and cell structure, as well as in secondary metabolic pathways that support growth and developmental processes [31,34]. Meanwhile, previous reports have also reported the influence of microgravity on antibiotic resistance mechanism of studied microorganisms [32,35]. Increased expression of genes associated with glucose metabolism in *Escherichia coli*; enhanced biofilm formation in *Klebsiella pneumoniae*, *E. coli*, and *Pseudomonas aeruginosa*; and increased virulence and stress resistance in *Salmonella enterica* under microgravity or simulated microgravity conditions have been reported in previous studies [31,32,33,34,35,36,37,38,39,40]. However, no effect on the production of the antibiotic peptide gramicidin in *Bacillus brevis*, no effect on the virulence of *Lactobacillus* species, and decreased virulence in *Streptococcus* and *Enterococcus* under microgravity conditions have also been reported, indicating the diverse and species-specific nature of microbial adaptation to microgravity [22,31,32,35,41,42]. In our previous study with *Acidovorax* sp., growth was higher under microgravity than under normal gravity; however, RNA-seq results showed that a greater number of genes were downregulated under microgravity, indicating that gene downregulation may be one of the adaptive strategies employed during exposure to microgravity [24].

Although *C. jejuni* is a microaerophilic microorganism commonly found in livestock and is a major foodborne pathogen, its behavior under microgravity conditions has not been previously investigated. The objective of this study was to determine how the simulated microgravity influences the growth, morphology, and virulence factors of *C. jejuni* NCTC 11168 (a virulent strain widely used in animal infection studies) [43].

## 2. Methods

### 2.1. Bacterial Strain and Culture Conditions

**Figure 1:**
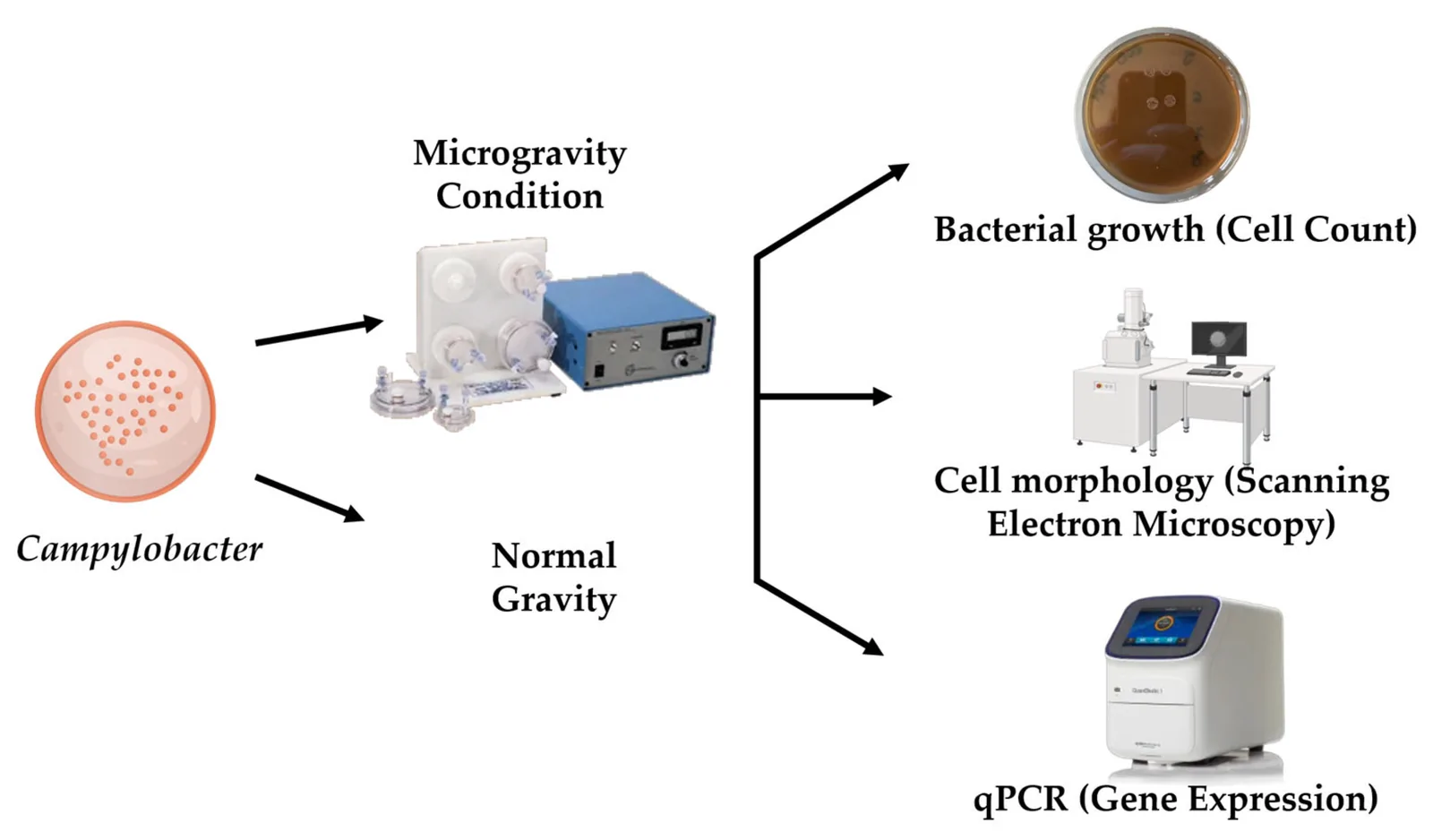
Schematic representation of the experimental design used in this study.

A schematic representation of the experimental design used in this study is presented in Figure 1. *C. jejuni* strain NCTC 11168 was cultured from stock culture (stored at −80 °C) on Mueller–Hinton agar (MHA, Criterion, Hardy Diagnostics, Santa Maria, CA, USA) plates supplemented with 5% laked horse blood (Hemostat laboratories, Dixon, CA, USA) and *Campylobacter* selective antibiotics (CCDA selective antibiotics, Himedia, Thane, India) for 48 h in microaerobic condition (5% O_2_ and 10% CO_2_, Heracell Vios 160i Tri-gas incubator, Thermo Fisher Scientific, Waltham, MA, USA) at 37 °C. Following incubation, colonies from each plate were scraped and transferred into 25 mL Mueller–Hinton Broth (MHB, Criterion, Hardy Diagnostics, Santa Maria, CA, USA) in conical flasks (150 mL). These liquid cultures were incubated for an additional 48 h in microaerobic conditions at 37 °C. After incubation, cultures were adjusted with fresh sterile MHB to an optical density of 0.01 (OD_600_) to create starting inoculums for all the following experiments. The inoculum was serially diluted with 1× phosphate-buffered saline (PBS; VWR International, Radnor, PA, USA) using tenfold serial dilutions (100 µL of inoculum added to 900 µL of 1× PBS), with dilutions continued through 10^−11^. Aliquots (10 µL) of each dilution were plated onto Mueller–Hinton agar (MHA) plates supplemented with 5% laked horse blood and incubated for 48 h under microaerobic conditions. Viable cells were enumerated and expressed as colony-forming units per milliliter (CFU/mL). This measurement was designated as the 0 h time point.

For simulated microgravity conditions, a Rotary Cell Culture System {(Synthecon Corporation, Houston, TX, USA); model RCCS-4 H; vessels: High-Aspect-Ratio Vessels [HARVs]} was used as described in our previous study, with a few modifications [24]. Because *Campylobacter* is microaerophilic, each autoclavable HARV chamber was sealed with an inner aluminum-foil lining to minimize oxygen diffusion and autoclaved before the bacterial inoculum was added. The HARV chambers were filled with prepared bacterial culture and sealed with an additional outer layer of Parafilm (microgravity [HARV]). This approach helped maintain a low-oxygen environment favorable to *Campylobacter* growth while allowing the HARVs to be operated in a conventional incubator for the simulated microgravity experiments and in an incubator shaker for the normal-gravity controls. For all experiments, inoculum preparation and initiation of the experimental conditions were completed within 30 min to minimize exposure to atmospheric oxygen. Although the gas composition within the vessels was not directly measured in each experiment, the same inoculum preparation, vessel-filling, sealing, and incubation procedures were followed consistently across all independent experiments and experimental conditions.

To simulate microgravity, the HARV chambers containing the bacterial suspension were rotated about a horizontal axis at 25 revolutions per minute (rpm), like in previous studies [24,28,29,30]. The RCCS provides a low-shear, low-turbulence culture environment in which the continuous rotation maintains cells in suspension and simulates aspects of microgravity. Based on the rotational radius of 21.59 mm, the corresponding rotational acceleration was calculated using the relative centrifugal force (RCF) equation to be approximately 0.015× *g* [24,28,29,30]. The RCCS was operated inside a forced-air incubator maintained at 37 °C.

For normal-gravity conditions, the HARV chambers were prepared as described previously, and 10 mL of bacterial suspension was loaded into each chamber (normal gravity [HARV]). The sealed HARV chambers were incubated at 37 °C in a conventional incubator shaker (Barnstead LAB-LINE MAX Q 4000, Barnstead International, Melrose Park, IL, USA) at 100 rpm. The shaker had a 19 mm orbital diameter, corresponding to an orbital acceleration of approximately 0.106 × g, and was used to maintain movement of the bacterial suspension under normal-gravity (1 g) conditions, as described in our previous report [24]. Under normal-gravity conditions, bacterial cultures were also prepared in 15 mL Falcon tubes (normal gravity [Falcon tubes]) to assess the effect of different vessel types on *Campylobacter* growth. Tubes were filled completely and sealed with Parafilm wrap. All chambers and tubes used for both normal gravity and microgravity were incubated in dark environments to minimize the influence of light exposure.

### 2.2. Viable Count Assessment

Growth and viability were assessed by determining colony-forming units (CFU/mL) at 0, 48, and 72 h. The experiments conducted at 48 h and 72 h incubation periods were performed as separate experimental series. Three independent biological replicate experiments were conducted, with at least two technical replicates per experiment. Tenfold serial dilutions were prepared in PBS, and aliquots were plated onto Mueller–Hinton agar (MHA) supplemented with 5% laked horse blood, as described previously. Plates were incubated for 48 h under the appropriate incubation conditions, after which, colonies were counted and CFU/mL was calculated. CFU counts from all biological replicates were included in the statistical analysis using an independent two-sample *t*-test assuming unequal variances (Welch’s *t*-test), with each biological replicate considered an independent experimental unit.

### 2.3. Electron Microscopy

**Figure 2:**
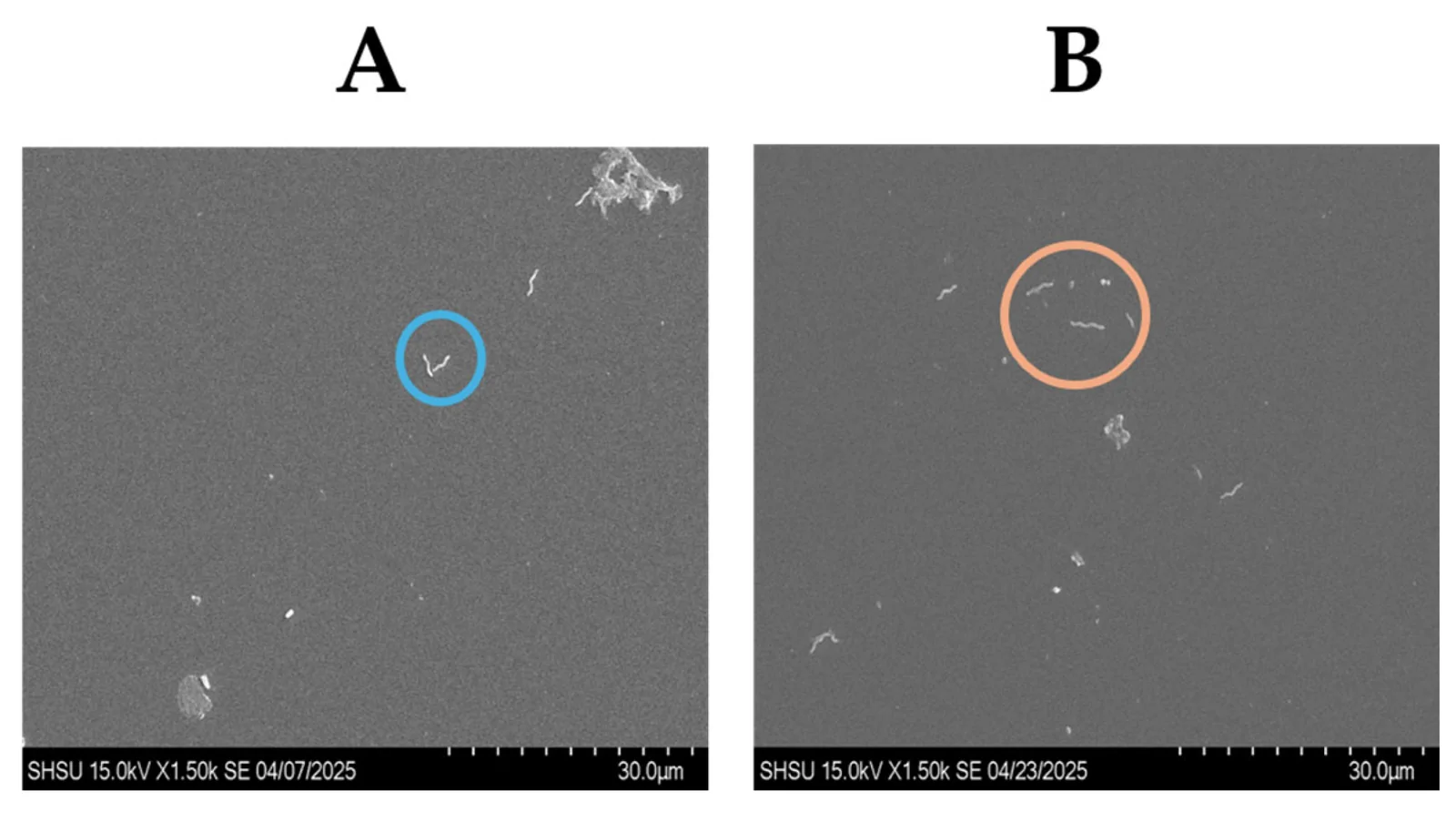
(**A**) Scanning electron micrograph of *Campylobacter* cells (highlighted inside blue circle) grown in simulated microgravity condition (48 h incubation). (**B**) Scanning electron micrograph of *Campylobacter* cells (highlighted inside orange circle) grown in normal gravity condition inside HARV chambers (48 h incubation).

For electron microscopy sample preparation, three biological replicate experiments were conducted independently, following similar protocol as described previously for the growth rate analysis. For each sample, 1 mL of bacterial suspension was collected after 48 h of incubation under three conditions (microgravity [HARV], normal gravity [HARV], and normal gravity [Falcon tubes]) and then centrifuged for 1 min at 10,000 rpm. The supernatant was discarded, and the cell pellet was fixed overnight with 2.5% glutaraldehyde in PBS. The cell pellets were subsequently washed three times with PBS and resuspended in PBS. A 10 µL aliquot of the bacterial cell suspension was spread onto multiple glass coverslips and air-dried for 30 min, followed by dehydration through a graded ethanol series (30%, 40%, 50%, 60%, 70%, 80%, 90%, and 100%). Gold coating and visualization of the dehydrated bacterial cells on the glass coverslips were performed at the Microscopy Center, Department of Biological Sciences, SHSU, following standard protocols [24]. All SEM images were acquired at 1500× magnification. For each condition, coverslips representing three biological replicates were randomly selected for acquisition of SEM images. Total 129 SEM images for microgravity, 160 SEM images for normal gravity in HARV chambers, and 87 SEM images for normal gravity in falcon tubes were acquired. SEM images were taken for the isolated cells for better cell-length measurement, as shown in Figure 2A,B as much as possible. Only clear images of *C. jejuni* cells exhibiting the typical morphology (Figure 2A,B) were included in the cell-length analysis. As many of the SEM images were included as possible to measure the lengths of at least 100 individual cells under each growth condition [microgravity {total 100 cells, replicate 1 = 32 cells, replicate 2 = 30 cells, and replicate 3 = 38 cells (mean cell length ± SD: 2.575 ± 0.674 µm, 2.192 ± 0.614 µm, and 2.092 ± 0.712 µm, respectively), normal gravity in HARV chambers {total 130 cells, replicate 1 = 62 cells, replicate 2 = 33 cells, and replicate 3 = 35 cells (mean cell length ± SD: 2.671 ± 0.898 µm, 2.578 ± 0.859 µm, and 2.781 ± 1.328 µm, respectively)}, and normal gravity in Falcon tubes {total 100 cells, replicate 1 = 30 cells, replicate 2 = 25 cells, and replicate 3 = 45 cells (mean cell length ± SD: 2.363 ± 0.862 µm, 1.892 ± 0.950 µm, and 2.522 ± 1.056 µm, respectively)}] using ImageJ (1.54p) [44,45]. For cell-length analysis, the distance from one end of the cell to the other was measured along with the cell image, with the cell’s curvature taken into consideration. The individual cell-length measurements from all biological replicates were used for mixed-effect analysis, and the mean cell-length values from each biological replicate (listed above for each set) were used for *t*-test: paired two samples for means.

### 2.4. Genomic Expression Analysis

After 48 h of incubation under simulated microgravity (HARV chamber) and normal-gravity (HARV chamber) conditions, 1 mL of bacterial culture was collected and mixed with 100 µL of pre-chilled stop solution (10% buffered phenol in ethanol) to preserve the RNA integrity. The samples were quickly vortexed and immediately frozen at −80 °C until RNA isolation. Experiment was conducted with triplicate biological replicates, and multiple samples were collected for each replicate to ensure sufficient RNA for subsequent analyses. For RNA isolation, the frozen samples were thawed at room temperature, and RNA was isolated using the Monarch Spin RNA Isolation Kit (New England Biolabs, Inc., Ipswich, MA, USA) according to the manufacturer’s protocol. The extracted RNA was treated with the Invitrogen TURBO DNA-free DNase Kit (Thermo Fisher Scientific, Waltham, MA, USA) to eliminate contaminating DNA from the RNA samples, following the manufacturer’s instructions. RNA concentration was measured using a NanoDrop spectrophotometer and the Qubit RNA BR Assay Kit (Life Technologies corporation, Eugene, OR, USA) with a Qubit 4 Fluorometer.

An equal amount of RNA (330 ng) was taken from each sample to be processed further for cDNA synthesis and qPCR analysis. cDNA synthesis was carried out using LunaScript RT SuperMix Kit (New England Biolabs), following the manufacturer’s instructions, in a 20 µL reaction. Five virulence genes for *Campylobacter* (listed in Table 1) were used to study the influence of variable gravity on gene expression [46]. The virulence-associated genes analyzed in this study were selected to represent key pathogenic functions, including fibronectin-binding and adhesion (*cadF*); invasion (*ciaB*); cytolethal distending toxin production (*cdtA*); AI-2-mediated quorum sensing and metabolic regulation (*luxS*); and flagellar motility (*flaA*), which encodes the major flagellin protein [46]. As an internal reference gene, the *16SrRNA* gene was included, like in previous studies [46,47,48,49,50,51,52,53], and the expression level of *16SrRNA* was found to be stable compared to other tested internal reference genes during our preliminary experiment. To determine the qPCR efficiencies for each reaction, a dilution series (10^1^–10^5^) was prepared from the cDNA template for each target gene. Standard curve slopes were calculated and used to determine the primers’ efficiency, like in a previous study that used these primers for gene expression analysis of virulence-associated genes in *Campylobacter* under oxidative stress [46]. The amplification factor for all primer pairs was 2.0, corresponding to an efficiency within the commonly accepted range of 90–110%. For qPCR analysis Luna Universal qPCR Master Mix Kit (New England Biolabs) was used. Each qPCR reaction had a total volume of 20 µL, consisting of 10 µL of qPCR master mix, 0.5 µL each of forward and reverse primers (10 µM) (Table 1), 8 µL of nuclease-free water, and 1 µL of cDNA template. Negative controls with no cDNA templates were also included. Cycling conditions for qPCR analysis were set on the Applied Biosystems Step One Real Time PCR system as follows: 1 min holding at 95 °C, and then 40 cycles of 15 s at 95 °C and 30 s for 60 °C. The change in gene transcription for each target gene was calculated using the 2^−ΔΔCT^ method [54]. qPCR assay was conducted at least twice for each sample with three technical replicates. Welch’s *t*-test was used for statistical analysis using independent biological replicate values.

### 2.5. Statistical Analysis

Statistical analysis and graphs were created using Microsoft Excel, GraphPad Prism Version 11, and R (4.6.1) (lem4 (2.0-6), lmerTest (3.2-1), and Emmeans (2.0.4) packages) as appropriate.

## 3. Results

### 3.1. Influence of Microgravity on C. jejuni Viable Count

**Figure 3:**
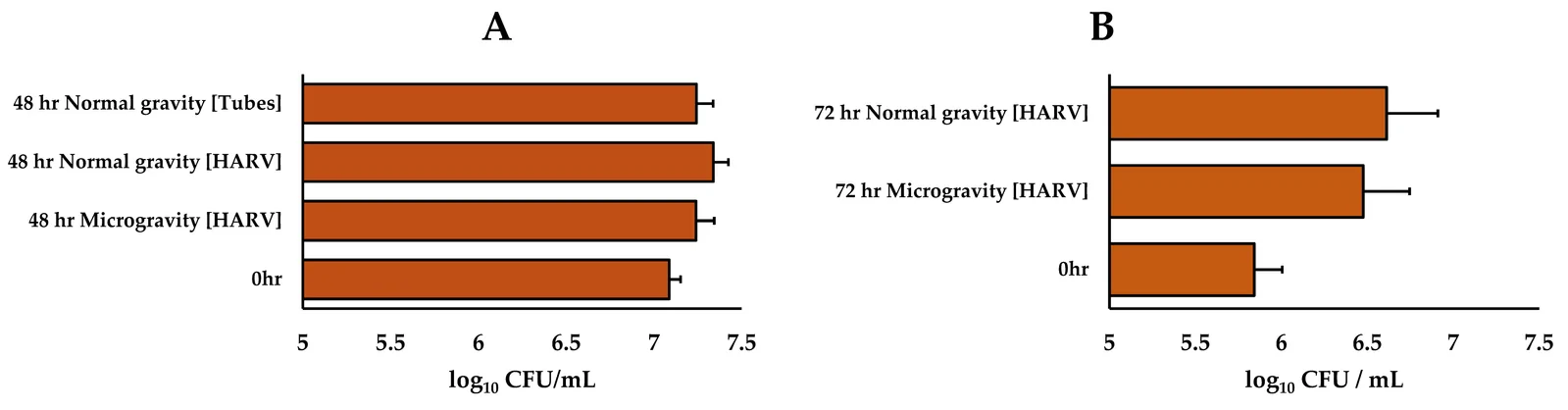
Survival or growth measurement of *C. jejuni* under normal conditions {(HARV, *n* = 6) and (Falcon tubes, *n* = 6)} and microgravity conditions (HARV, *n* = 6) (**A**) at 48 h and (**B**) 72 h, both showing no significant difference. Error bars represent the standard deviation.

Although *C. jejuni* growth was lower under simulated microgravity than under normal-gravity conditions at both incubation times (48 and 72 h) (Figure 3), suggesting that microgravity may negatively affect *Campylobacter* growth, the differences were not statistically significant. At 48 h, the mean log CFU/mL was 7.241 ± 0.103 (mean ± SD) under simulated microgravity and 7.338 ± 0.086 under normal-gravity conditions in HARV chambers; however, the difference was not statistically significant (two-tailed *t*-test, *p* = 0.105). Similarly, growth under simulated microgravity did not differ significantly from that under normal-gravity conditions in Falcon tubes (mean log CFU/mL = 7.243 ± 0.095; two-tailed *t*-test *p* = 0.977). Growth of *C. jejuni* under normal-gravity conditions in HARV chambers versus Falcon tubes was also not significantly different (two-tailed *t*-test, *p* = 0.096), indicating that vessel type did not significantly influence bacterial growth. At 72 h, growth under simulated microgravity (mean log CFU/mL = 6.477 ± 0.269) was not significantly different from that under normal-gravity conditions in HARV chambers (mean log CFU/mL = 6.612 ± 0.298; *p* = 0.515).

### 3.2. Influence of Microgravity on Cell Length

Scanning electron microscopy (SEM) revealed differences in the cell length of *C. jejuni* grown under simulated microgravity and normal-gravity conditions (Figure 2). Cells grown under simulated microgravity in the HARV chamber had a mean cell length of 2.286 ± 0.065 µm (mean ± SD; calculated from the mean cell length of three biological replicates), compared with 2.677 ± 0.010 µm for cells grown under normal-gravity conditions in the HARV chamber. Cells grown under normal-gravity conditions in Falcon tubes had a mean cell length of 2.259 ± 0.107 µm. Thus, based on the mean cell length calculated for each biological replicate, cells grown under simulated microgravity appeared shorter than those grown under normal-gravity conditions in the HARV chamber, whereas cells grown under normal-gravity conditions in Falcon tubes were also shorter than those grown under normal-gravity conditions in the HARV chamber.

A paired two-sample *t*-test using the mean cell length from each of the three biological replicates did not detect statistically significant differences between any of the groups (normal gravity [HARV] vs. simulated microgravity [HARV], *p* = 0.1498; normal gravity [HARV] vs. normal gravity [Falcon tubes], *p* = 0.0904; simulated microgravity [HARV] vs. normal gravity [Falcon tubes], *p* = 0.9156).

To account for the large number of individual cell-length measurements while accounting for variation among biological replicates, a linear mixed-effects model was fitted with experimental condition as a fixed effect and biological replicate as a random effect. Analysis of individual cell-length measurements showed that cells grown under simulated microgravity in the HARV chamber were significantly shorter than cells grown under normal-gravity conditions in the HARV chamber (2.276 ± 0.486 µm vs. 2.677 ± 1.034 µm, *p* = 0.0052). Cells grown under normal-gravity conditions in Falcon tubes were also significantly shorter than those grown under normal-gravity conditions in the HARV chamber (2.317 ± 1.008 µm vs. 2.677 ± 1.034 µm, *p* = 0.0130). No statistically significant difference was detected between cells grown under simulated microgravity in the HARV chamber and those grown under normal-gravity conditions in Falcon tubes (*p* = 0.9641). In a subsequent analysis excluding the normal-gravity (Falcon tube) data, a linear mixed-effects model was fitted to the normal-gravity (HARV) and simulated-microgravity (HARV) datasets, with gravity condition as a fixed effect and biological replicate as a random effect. The model indicated a statistically significant effect of gravity condition on cell length (*p* = 0.0013).

### 3.3. Influence of Microgravity on Genomic Expression of Targeted Virulence Genes

Gene expression analysis showed apparent upregulation of *cadF* and *cdtA* under simulated microgravity (Table 2). However, none of these changes were statistically significant when compared to normal-gravity conditions. Overall, microgravity did not produce measurable differences in the transcriptional levels of the tested virulence genes.

### 3.4. Discussions and Conclusions

Microgravity is known to impose unique stress on bacterial organisms [31]. This report is the first one to study the influence of simulated microgravity on *Campylobacter* physiology and morphology. In our study, *C. jejuni* exhibited a comparatively lower cell count than normal gravity condition at both 48 h and 72 h incubation periods. However, the result was not significant. Meanwhile, SEM analysis revealed that *C. jejuni* cells grown under simulated microgravity appeared shorter than those grown under normal gravity conditions in the HARV chambers. However, cells grown under normal gravity conditions in Falcon tubes were also shorter than those grown in the normal gravity HARV chambers. The differences in mean cell length were not statistically significant based on the paired *t*-test analysis but were significant in mixed effect analysis of individual cell-length measurements. Therefore, caution is warranted when directly attributing the observed reduction in cell length to simulated microgravity. In particular, differences in vessel configuration, fluid dynamics, and rotational acceleration between the RCCS and conventional incubator shaker may have influenced the observed cellular phenotype.

Morphological changes in microgravity are often linked to alterations in membrane structure and cell envelope integrity, which can occur when sedimentation and fluid shear are minimized [56]. In microgravity, fluid transport, sedimentation, and diffusion change [31,33]. The materials are floating in space as the machine rotates, and diffusion is the driving factor that allows movement in the media. Therefore, the spreading of nutrients and oxygen is much slower, and the accumulation of carbon dioxide happens faster [33]. Limitation of cellular transport during microgravity condition might negatively impact cellular growth [57]. The cells may settle into creating a shorter lag phase, and the change can be the reason the bacterial length was shorter in microgravity [33]. Simulated microgravity condition could induce stress response in *E. coli* strain in previous studies [58,59]. In contrast, our own previous study found higher growth of *Acidovorax* sp. 1,608,163 under simulated microgravity than under normal gravity condition [24]. A similar result of higher bacterial growth of *E. coli* under microgravity was reported previously [59]. Hence, the influence of microgravity might differ among bacterial species under microgravity condition, as noted in previous reports [21,31].

Five virulence-associated genes (*cadF, ciaB, cdtA, luxS,* and *flaA)* were examined to determine whether microgravity could influence the pathogenicity of *Campylobacter*. Although microgravity may impose stress on *Campylobacter*, the microgravity conditions did not significantly alter the expression of these genes. In a separate previous study, the expression of *C. jejuni* virulence genes was influenced by environmental stress, such as oxidative stress [46]. However, in our study, microgravity conditions did not significantly alter the expression of the five selected virulence genes. To our knowledge, no published studies have investigated *Campylobacter* infection or pathogenesis under microgravity conditions. Previous studies have reported variable effects of microgravity on bacterial virulence, including attenuation in some bacterial species and enhancement of virulence in others [57,60]. Likewise, variable effects on secondary metabolite production, including antibiotic resistance, have been documented [57].

This study indicated that simulated microgravity had limited influence on *Campylobacter* during short-term incubation, with no statistically significant differences in viable cell counts (48 h and 72 h incubation), cell length (48 h incubation), or the expression of the selected virulence associated genes (48 h incubation). However, the observed trends toward shorter cell length and lower viable counts at both incubation times warrant further investigation. We hypothesize that simulated microgravity may produce more pronounced effects on *Campylobacter* physiology and survival during prolonged incubation, which should be evaluated in future studies using extended incubation periods. Likewise, a whole-genome expression analysis, such as RNA sequencing (RNA-Seq), may provide further insights into the response of *Campylobacter* to microgravity.

## Figures and Tables

**Table 1:** Primers used in this study for qPCR analysis of selected virulence genes [46,55].

Gene		Primer Sequence
*cadF*	F	TTC TAT GGTTTA GCA GGT GGA G
	R	TTA CAC CCG CGC CAT AAT
*ciaB*	F	AAA AGC TTG GCA AGA AGC TG
	R	ATG CCA CCG CAT GAG TAT AA
*cdtA*	F	GGA TTT GGC GAT GCT AGA GTT
	R	CAT TTG TGC GTG ATT GCT TG
*luxS*	F	AAA ATG CCA GCT CCT GCT GT
	R	GTG CGA CAA CCC ATA GGT GA
*flaA*	F	GGA TGG CGA TAG CAG ATA GTT T
	R	CTC ATC CAT AGC CTT ATC AGC A
*16SrRNA*	F	TGC TAG AAG TGG ATT AGT GG
	R	GTA TTA GCA GTC GTT TCC AA

**Table 2:** Gene expression analysis (qPCR assays) for 5 selected virulence genes of *C. jejuni* strain under simulated microgravity vs. normal gravity condition. Table represents the Log2 Fold Change analysis from the qPCR assay and statistical analysis (*n* = 3).

Gene	Mean ΔCt Microgravity	Mean ΔCt Normal Gravity	Log2FoldChange	Standard Deviation	Welch *t*-Value	*p*-Value	95% CI for Log_2_FC
*cadF*	1.706	2.973	1.266	1.768	−1.123	0.3474	−2.439 to 4.972
*ciaB*	1.413	1.475	0.062	3.8	−0.02	0.9849	−8.463 to 8.586
*cdtA*	2.228	4.357	2.13	1.399	−2.051	0.2538	−6.759 to 11.018
*flaA*	2.889	2.539	−0.35	1.936	0.269	0.8421	−7.077 to 7.986
*luxs*	4.544	4.999	0.455	3.385	−0.221	0.8043	−4.308 to 3.607

## Data Availability

The original contributions presented in this study are included in this article. Further inquiries can be directed to the corresponding author.
